# A clinical audit of maternal syphilis in a regional hospital in KwaZulu-Natal, South Africa

**DOI:** 10.4102/sajid.v35i1.115

**Published:** 2020-04-30

**Authors:** Onankoy A. Onyangunga, Thajasvarie Naicker, Jagidesa Moodley

**Affiliations:** 1 Optics and Imaging Centre, School of Laboratory Medicine and Medical Sciences, University of KwaZulu-Natal, Durban, South Africa; 2 Women’s Health and HIV Research Group, Department of Obstetrics and Gynaecology, School of Clinical Medicine, University of KwaZulu-Natal, Durban, South Africa

**Keywords:** Maternal syphilis, Congenital syphilis, HIV, RPR, Reverse algorithm

## Abstract

**Background:**

Despite the availability of screening guidelines and effective treatment for maternal syphilis (MS), its prevalence remains high and is re-emerging in many parts of the world. This might be because of varying screening tests and algorithms for the laboratory diagnosis and treatment of syphilis. In addition, HIV co-infection may compromise the elimination of MS. The present study is a clinical audit of the prevalence of MS in KwaZulu-Natal, South Africa, using the ‘Traditional Algorithm’ screening.

**Methods:**

This was a retrospective audit in which data on syphilis testing were obtained over a 1-year period (2016) at a large regional hospital in South Africa. The standard screening test at the study site was the non-treponemal antigen, rapid plasma reagin (RPR). Data on the prevalence of MS and comorbidity with HIV infection were analysed.

**Results:**

There were 10 680 deliveries in the study period of which 118 were RPR reactive, giving an MS prevalence of 1.1%. MS occurred predominantly in the age groups < 18 and > 35 years (*p* = 0.001). The prevalence of HIV infection was 41.2% (*n* = 4451). Seventy-two (61.0%) had both HIV and MS infection, whilst 46 (39.0%) had discordant results (*p* = 0.001).

**Conclusion:**

We report an increase in the prevalence of MS compared to previous South African National Antenatal Syphilis Surveillance studies. This may be because of the prozone effect caused by HIV infection on the sensitivity of the RPR. We propose a change in MS screening, using a Rapid DUO (Dual HIV and syphilis point of care test) and Reverse Algorithm for screening that could improve the sensitivity, detection and management of both diseases.

## Background

The World Health Organization (WHO) estimates that between 1.36 and 2.0 million pregnancies are affected by syphilis each year.^1^ Africa has the highest incidence of syphilis with 63% of cases being reported annually in pregnant women.^1,2^ The incidence of maternal syphilis (MS) and congenital syphilis (CS) is reported to have decreased to 38% and 39% in 2008 and 2012 respectively.^3,4,5^ However, there is a recent concern about the re-emergence of syphilis, following reports of the increasing incidence of MS and CS rates in the United States, Eastern Europe and Russia.^6,7,8^ Regarding Southern Africa, in Swaziland and Mozambique, during the period 2009–2010, antenatal clinic attendees had MS prevalence rates of 5.7% and 8.3% respectively, whilst South Africa, Namibia, Lesotho and Botswana had MS prevalence rates of 1.3% – 2.2%.^9,10,11,12^ In 2015, the South Africa National Surveillance Reports (NASHSPS) found that the prevalence of MS had increased to 2.0% after a slight drop from 1.9% to 1.6% in 2011.^13,14^ It is noteworthy that the prevalence of MS between the two NASHSPS reports for 2008–2011 and 2015, however, found mixed trends in different provinces;^13,14^ in particular, KwaZulu-Natal (KZN) and the Free State had increased MS prevalence rates from 0.4% to 2.3% and from 1.9% to 4.6% respectively, whilst Gauteng, the Northern Cape and Western Cape had registered declines in the 2015 report.^13,14^

Among pregnancies affected by MS, 50% – 80% result in adverse birth outcome with inadequate treatment.^3,14^ These adverse outcomes can be avoided by repeated testing during pregnancy to detect new cases with consequential appropriate treatment and better management during pregnancy.^15^ Taking cognisance of the increasing global prevalence of MS, researchers have suggested that health authorities need to review and strengthen their policies for screening and treating syphilis during pregnancy and have indicated that the results of screening tests are probably dependent on the type of tests used. They have also indicated that comorbid conditions such as HIV infection and malaria may impact negatively on the screening test utilised.^16,17,18,19^

The WHO has recommended guidelines for screening of syphilis during pregnancy.^20^ Nonetheless, the implementation of these guidelines is adapted to the resources and specific condition of each country. Factors such as accessibility, transport to the healthcare centre, healthcare providers and laboratory facilities are determinants for a successful screening programme. According to National SA Guidelines, screening for syphilis during pregnancy in the public sector involves a cost-effective non-treponemal test, rapid plasma reagin (RPR) followed by a confirmatory treponemal test, the treponemal pallidum hemagglutination assay (TPHA).^21^ Despite its low sensitivity and specificity, the RPR is the most cost-effective screening test utilised worldwide.^22,23,24^ The RPR test measures the reactivity of non-specific treponemal antibodies, both IgM and IgG of sera from patients with syphilis to non-specific cardiolipin-cholesterol-lecithin antigens.^22,23,24^ The limitations of RPR are false-positive reactivity, particularly in infectious diseases such as malaria and HIV infection that have an impact on the ‘prozone phenomenon’. In prozone effect, excess of antibodies of different origins, including those of *Treponema pallidum*, is observed in 1% – 2% of cases with secondary syphilis.^22,23,24^ False-negative results associated with RPR test have no clear explanation and authors recommend the use of the TPHA test, particularly in HIV-positive patients.^25^ More recently, it has been suggested that screening for syphilis during the antenatal period should be carried out, using the rapid immune-chromatographic tests that are effective in resource-constrained settings and can be adapted by the Point of Care (POC) philosophy.^26^ These tests, however, still have limitations about their sensitivity, specificity and cost-effectiveness, and thereby affecting the prevalence rates of syphilis. Furthermore, the stage of disease often affects the sensitivity of both non-treponemal and treponemal tests.^22,23,24,26,27^ Among the infective co-morbidities with syphilis in pregnancy, HIV infection is the commonest in Southern and Eastern Africa. National prevalence rates of syphilis and HIV per year across 11 countries of Southern and Eastern Africa, show a strong positive association between the pre-peak HIV and the peak HIV infection prevalence at the country level.^28,29,30,31,32^ It has also been shown that MS can increase HIV transmission by two- to threefold *in utero* or at delivery.^30^

In South Africa, the National Antenatal Sentinel HIV and Syphilis Prevalence Survey (2011) reported low prevalence rates of syphilis in KZN, the province with the highest HIV burden.^13,14^ Manda et al. (2012) considered that the discordant ecological and spatial effects between HIV infection and syphilis may indicate that syphilis is not suited as a predictor of HIV prevalence, but concluded that the trends suggest that syphilis control and prevention programmes have been successful.^33^ These results are, however, disparate with recent concern about rising rates of syphilis in the United States.^6,7,8^ The latest NASHSPS report shows a dramatic increase in HIV infection and MS prevalence of 7.0% and 1.9% respectively.^14^ Therefore, in view of these concerns it is important to review the screening methods, the target population groups and to identify the factors influencing the prevalence of MS and CS. The present audit evaluates the prevalence of MS at a large regional hospital in Durban where the traditional screening algorithm for MS testing is utilised.

## Methods

After hospital regulatory permission, a retrospective clinical data analysis was carried out at the antenatal clinic of a regional hospital in the south of Durban, KZN. This hospital conducts about 12 000 deliveries a year and serves a lower socioeconomic urban population. The labour ward and postnatal medical case file registries were used to retrieve the appropriate data.

The standard practice at the hospital was that all mothers are counselled and given information about antenatal serological testing at the first antenatal visit. Screening tests, included a syphilis, HIV infection and rhesus blood group testing performed either during the antenatal period or before discharge from hospital in women who had not attended antenatal care. All women received counselling and HIV testing.

The standard screening for MS procedure was to send a blood sample to the National Health Laboratory Services of South Africa to perform the non-treponemal syphilis (RPR) test. The test was considered syphilis positive at dilution of ≥ 1:8. A confirmatory TPHA test was needed for dilutions < 1:8. Whilst awaiting the TPHA result, the mother was treated with penicillin, as she was considered ‘syphilis exposed’. In case of the TPHA being negative, the treatment was discontinued. A rapid non-treponemal test was used in some clinics; if positive, the pregnant women were treated on-site and the RPR algorithm described, served as a follow-up protocol.

Treatment consisted of 2.4 M IU bicillin (long-acting penicillin) intramuscular injections weekly for three consecutive weeks; in addition, women were counselled and advised about partner treatment. Furthermore, the national guidelines on testing for MS require all negative RPR pregnant women to be retested at 32 weeks.

At the study site, the neonatal unit practised the policy of providing full treatment to all ‘syphilis-exposed’ neonates, and both the neonate and mother were requested to return for the 6 weeks post-delivery follow-up visit. Rapid HIV testing, according to national guidelines, was carried out for all pregnant women and all HIV-infected women were treated with HAART.^21^ All HIV-uninfected women had repeat testing at 3 months after the initial test or at delivery, or immediately postpartum.

## Statistics

Statistical data analyses were conducted, using GraphPad Prism5 Software. The mean ± standard deviation (s.d.) of each group study was calculated. Prevalence testing was used for syphilis and HIV infection in the study population, and non-parametric tests were utilised to compare categorical data. A *p*-value of > 0.05 was considered statistically significant.

### Ethical consideration

This article followed all ethical standards for carrying out research, with protocol reference number: BREC/00001072/2020, from the Biomedical Research Ethics committee, University of KwaZulu-Natal, South Africa.

## Results

### Maternal syphilis

There were 10 680 deliveries during the study period; 118 women (1.1%) tested syphilis RPR positive.

### Congenital syphilis

There were no cases of symptomatic CS recorded and all ‘exposed neonates’ (*n* = 118) had a full course of penicillin treatment, according to the hospital clinical protocol.

### Human immunodeficiency virus co-infection

The HIV-infected prevalence rate was 41.7% (*n* = 4451); in addition, 2457 HIV-uninfected mothers were retested during the antenatal period and 59 (1.2%) seroconverted. At delivery, from 3762 women 57 had seroconverted, resulting in a seroconversion rate during pregnancy of 1.5%.

### Age groups in the maternal syphilis-positive group

The mean age s.d. of the MS group was 26.0 ± 6.68 years. The prevalence of MS, according to age groups, is shown in Table 1. The prevalence of MS varied from 0.7% to 1.4%. The demographic data showed that MS occurred in all age groups with a relative predominance in the age group ≤ 18 years (1.4%) and in the age group ≥ 35 years (1.3%) with a significantly low prevalence (0.7%) for age group 30–34 years (*p* = 0.001).

**TABLE 1:** Age group of mothers, maternal syphilis and human immunodeficiency virus co-infection.

Age group	MS	RPR+/HIV-	RPR+/HIV+	Deliveries	Prevalence of MS (%)
≤ 18 years	12	10	2	850	1.4
19–24 years	45	19	26	3986	1.1
25–29 years	34	11	23	2932	1.2
30–34 years	13	3	10	1840	0.7
≥ 35 years	14	3	11	1072	1.3
**Total**	**118**	**46**	**72**	**10 680**	**1.1**

MS, maternal syphilis; RPR, rapid plasma reagin; HIV, human immunodeficiency virus; +, positive (infected); -, negative (uninfected).

### Syphilis and human immunodeficiency virus co-infection

There were 72 (66.1%) concordant cases (RPR reactive and HIV-infected) representing 1.6% of HIV-infected mothers, 46 (39.0%) cases were discordant (RPR reactive and HIV negative) representing 0.7% of HIV-uninfected. There was a significant difference in the number of the concordant and discordant groups (*p* = 0.001), including age (25.0 ± 5.1 years vs. 28.1 ± 6.8 years; *p* = 0.0001). In other words, a HIV-infected pregnant woman had a twofold risk of having syphilis.

## Discussion

The present clinical audit demonstrates an increase in the MS prevalence and absence of symptomatic CS. Furthermore, we report a twofold risk increase of MS co-infected with HIV in pregnant women.

These results highlight the need for a special screening strategy of HIV-uninfected pregnant women within this high HIV endemic zone of South Africa. There is also the need to strengthen the screening and treatment of syphilis in this geographical catchment area.

The current MS prevalence rate at the study site was higher than the 0.4% reported by the NASHSPS in 2011 for the province of KZN^13^ and lower than the 2.3% reported in 2015,^14^ showing an increase of almost threefolds and sevenfolds respectively, but remains low in absolute values compared to the national average prevalence in 2011 (1.5%) and 2015 (2%) and to the KZN prevalence rate (2.3%) reported in the 2015.^14^

The prevalence of syphilis at our study site was lower than the average for the eThekweni district among the lowest in KZN districts.^13,14^ Importantly, our results are corroborated by other studies^1,2,6,7,8^ that report a surge of MS worldwide.^1,2,6,7,8,14^

The MS prevalence in the present study raises the possibility that the MS prevalence rate is underestimated. This hypothesis may be true, as the RPR test used for screening of syphilis during pregnancy has limitations mainly because of its poor sensitivity and specificity.^22,23,24^ The specificity of the RPR has shown a high false-negative rate of 11% compared to the treponemal test (TPHA) in a recent study conducted in Alexandra Township-Gauteng, South Africa.^9^ Indeed, different sensitivities and specificities have been reported about syphilis tests in the literature.^22,23,24^

Despite controversies on diagnostic tests, *T. pallidum* remains highly responsive to penicillin administration, making syphilis a curable infectious disease. *Treponema pallidum* is also sensitive to other antibiotics used in the treatment of sexually transmitted infections (STIs) and common respiratory infections.^9,21,34^ It is possible that HIV-infected patients, already on treatment, get a plethora of antibiotics for common infectious conditions, and therefore undetected syphilis gets unwittingly treated and its prevalence reduced. In addition, the overuse of antibiotics by general practitioners and primary healthcare clinics may also inadvertently be treating syphilis and hence contributing to the low syphilis rates in comparison to the previous high rates reported in the last decade.^1,3^

Congenital syphilis is predominantly asymptomatic. Up to 60% of infected neonates do not have signs at birth and require high suspicion in the exposed neonates group.^34,35^ As the exclusion of CS clinically and with serum diagnosis remaining difficult, the step to treat all exposed neonates seems acceptable in a setting in which a follow-up 6 weeks postnatal visit is poor.

The prevalence of syphilis in HIV-infected pregnant women may be the result of interference between the RPR test and serum of HIV-infected patients. The co-infection is common and has been described in previous reports.^16,17,29,30,31,32^ Some reports have linked the decline of syphilis and MS to the high mortality because of HIV or AIDS and syphilis re-emergence, and the rise in its prevalence was associated with the introduction of antiretroviral therapies (ARVs).^31,32^ However, the ARVs have yet not been reported to affect the prevalence of syphilis.^32^

It should be also noted that the South African antenatal HIV and syphilis survey considers only the seropositive antenatal attendees at their first visit and does not consider the possibility of seroconversion as one of plausible explanations for the low prevalence of syphilis. The present study also observed a significant number of HIV seroconversions during the antenatal period and at delivery, and, as a fact, cannot rule out the possibility of syphilis seroconversion. The seroconversion among the pregnant women has been reported previously. Lawi et al. (2015) in Tanzania^12^ noted a seroconversion of syphilis and HIV at respectively, 2.7% and 2.0% with overall prevalence of 2.3% and 7.2% for syphilis and HIV infection. The absence of repeat testing during pregnancy and at delivery may also contribute to a low or underestimated prevalence of MS.

The co-infection of syphilis and HIV is not well understood, but a decreased of CD4 counts and enhanced viral load in an HIV-infected population has been reported in patients with syphilis.^32,33^ Furthermore, RPR and treponemal test have shown false-positive and false-negative results, and syphilis results need to be regarded with caution in a HIV endemic setting. Indeed, González-López (2009)^34^ observed that HIV patients have a slow immune response to syphilis treatment and suggest that it may influence the result of the RPR. The mechanism through which this happens is still unclear and could involve immunoglobulin abnormalities, cardiolipin lecithin antigen, polyclonal gammopathy, protein and cholesterol. The use of antiretroviral seems to give false RPR positive, particularly in inflammatory conditions such as HIV.^32,34,35^ It is possible that non-treponemal tests like the RPR gives false-negative results in HIV-positive patients; therefore, further treponemal tests should be recommended.^22,23,24^

There were mixed results shown by the NASHSPS 2015 study with a decrease on MS in some provinces (Gauteng, Northern Cape and Western Cape) with concomitant decrease or increase of HIV among women attending ANC.^14^ The re-emergence of syphilis in KZN province (from 0.4% to 2.3%) coincides with the increase of HIV prevalence (7%) and warrants further investigation.

In the present study, the observations showing a significant increase of MS in both ≤ 18-year-old age group and ≥ 35-year-old age group compared to 30–34-year-olds contrast with the national 2015 report.^14^ A finding of 12.9% of MS in the ≥ 35-year age group is difficult to explain given that the frequencies of STIs are reported to be higher in adolescences and young adults (below 34 years).^14^ This also needs further investigation.

The present study has limitations. It was a retrospective chart review and not all records may have been fully documented. Furthermore, it seems that the study site was not performing or not recording the results of repeated syphilis screening test at 32 weeks and in the postpartum period to evaluate the efficiency of treatment and re-infection as required by the national guidelines.^21^ The scenario of providing complete treatment to the exposed neonates not having had follow-up serological testing in the post-partum is another limitation.

In view of the results of the present study and looking at the severe comorbidity of HIV and syphilis, the authors recommend the use of ‘Reverse Algorithm’ for the HIV-positive mothers.^36,37^ The DUO rapid testing at POC can improve the strategies and accelerate the elimination of CS and HIV infection.^36,37,38,39,40^

However, the choice of any specific test may depend on the cost and its ‘user capabilities’ for the healthcare providers.^39^

## Conclusion

This study has confirmed the increase of MS and its predominance among HIV-infected pregnant women in KZN. The elimination of MS and CS can only be achieved by the reduction of HIV prevalence. A DUO (HIV/syphilis), cost-effective and user-friendly testing is then proposed as a screening test towards the further reduction of maternal morbidity and elimination of mother-to-child transmission of both syphilis and HIV. This study calls for further analysis on the maternal and CS nationwide and in KZN.
